# Focused ultrasound for blood–brain barrier opening in brain tumor patients: Technical nuances

**DOI:** 10.1093/noajnl/vdag020

**Published:** 2026-01-31

**Authors:** Massimiliano Del Bene, Valentina Caldiera, Mario Stanziano, Giovanni Carone, Alessandro Falanga, Annica Piccardi, Giorgia Simonetti, Carla Carozzi, Giulia Frazzetta, Antonio Silvani, Francesco Prada, Marina Grisoli, Elisa Ciceri, Francesco DiMeco

**Affiliations:** Department of Neurosurgery, Fondazione IRCCS Istituto Neurologico Carlo Besta, Milan, Italy; Department of Experimental Oncology, IEO, European Institute of Oncology IRCCS, Milan, Italy; Department of Pharmacological and Biomolecular Sciences, University of Milan, Milan, Italy; Department of Neuroradiology, Fondazione IRCCS Istituto Neurologico Carlo Besta, Milan, Italy; Department of Neuroradiology, Fondazione IRCCS Istituto Neurologico Carlo Besta, Milan, Italy; Department of Neurosurgery, Fondazione IRCCS Istituto Neurologico Carlo Besta, Milan, Italy; Department of Neurosurgery, Fondazione IRCCS Istituto Neurologico Carlo Besta, Milan, Italy; Department of Neurosurgery, Fondazione IRCCS Istituto Neurologico Carlo Besta, Milan, Italy; Neuro-Oncology Unit, Fondazione IRCCS Istituto Neurologico Carlo Besta, Milano, Italy; Neurointensive Care Unit, Department of Neurosurgery, Fondazione IRCCS Istituto Neurologico Carlo Besta, Milan, Italy; Department of Treatment Interventions, Insightec Europe, Milan, Italy; Neuro-Oncology Unit, Fondazione IRCCS Istituto Neurologico Carlo Besta, Milano, Italy; Department of Neurosurgery, Fondazione IRCCS Istituto Neurologico Carlo Besta, Milan, Italy; Acoustic Neuroimaging and Therapy Laboratory, Fondazione IRCCS Istituto Neurologico Carlo Besta, Milan, Italy; Department of Neurological Surgery, University of Virginia Health System, Charlottesville, Virginia, USA; Focused Ultrasound Foundation, Charlottesville, Virginia, USA; Department of Neuroradiology, Fondazione IRCCS Istituto Neurologico Carlo Besta, Milan, Italy; Department of Neuroradiology, Fondazione IRCCS Istituto Neurologico Carlo Besta, Milan, Italy; Department of Oncology and Hematology-Oncology, Università Degli Studi di Milano, Milan, Italy; Department of Neurological Surgery, Johns Hopkins Medical School, Baltimore, Maryland, USA

**Keywords:** blood–brain barrier opening, brain tumor, focused ultrasound, protocol, therapeutic ultrasound

## Abstract

**Introduction:**

Focused ultrasound (FUS) is making rapid advances in the field of neurological sciences. While proven effective for tremor, pain and dystonia, FUS hold promises in experimental applications like blood–brain barrier opening (BBBO). BBBO is an emerging non-invasive option for intracranial brain tumor treatments by enhancing drug delivery and enabling liquid biopsies. Clinical trials underscore the need for consistent use of FUS for BBBO, which requires sophisticated facilities and standardized protocols to ensure efficacy and reproducibility. This manuscript outlines a single-center experience using MR-guided FUS (MRgFUS) for BBBO in patients with glioblastoma, incorporating technical insights and describing the evolution of a workflow mirroring an ongoing learning curve.

**Methods:**

We developed an optimized protocol that includes comprehensive pre-procedural evaluations, a patient preparation process designed to minimize discomfort, and a sedation protocol utilizing dexmedetomidine, presented as a technical note involving 4 patients. The procedures were conducted using the Exablate Neuro 4000 Type 2 system, with MRI providing precise targeting and monitoring.

**Results:**

Our optimized protocol significantly enhanced patient comfort and reduced procedure times. Employing a large treatment envelope and a 64-spot grid allowed for near-total brain coverage, minimizing the effort for pre-planning and reducing sonication time. Optimized MRI protocol contributed to the reduction in duration and confirmed successful BBBO, indicated by specific imaging signs and the resolution of these effects without any clinical complications.

**Conclusion:**

The protocol we developed for MRgFUS-BBBO offers substantial improvements in terms of procedure efficiency, imaging quality, and patient comfort. This approach could be adopted in other FUS treatment settings to optimize workflows, reduce procedure times, and improve patient outcomes.

Key Points

- Optimized MRgFUS-BBBO protocol enhances efficiency and patient comfort.
- 64-spot grid improved sonication efficiency, enabling larger treatment volumes.

Importance of the Study

Focused ultrasound (FUS)-mediated blood–brain barrier opening (BBBO) represents a promising yet technically complex approach for enhancing drug delivery and enabling liquid biopsies in brain tumor patients. This study provides a detailed account of the evolution of an MR-guided FUS (MRgFUS) workflow, incorporating critical technical refinements that improve procedural efficiency, patient comfort, and imaging quality. Our findings demonstrate significant reductions in procedure time, enhanced sonication coverage, and an optimized sedation protocol, all contributing to increased feasibility and reproducibility. Compared to prior literature, our experience highlights the rapid learning curve associated with MRgFUS-BBBO and the importance of standardized protocols to maximize clinical benefits. These insights hold translational significance for broader implementation in clinical trials and future therapeutic applications, ultimately advancing the role of FUS in neuro-oncology.

Focused ultrasound (FUS) technology is undergoing exponential advancement in the field of neurological sciences, proving to be a versatile and effective tool in a range of applications.^1^ These encompass a range of recognized clinical applications, including the treatment of tremors and pain, as well as still experimental applications, such as blood–brain barrier opening (BBBO), and promising pre-clinical applications, including histotripsy and neuromodulation.^2^^,^^3^ Of particular interest is the prospective for non-invasive treatment of brain tumors, in particular, relying on BBBO.^4^ A substantial body of evidence from numerous studies has demonstrated the feasibility of inducing BBBO as a means of improving the penetration of various chemotherapeutic agents into the central nervous system and augmenting the yield of biomarkers in the blood of brain tumor patients. This evidence has introduced the concepts of FUS-enhanced therapy and liquid biopsy.^5^ Nowadays, a growing body of clinical trials is supporting the consistent clinical implementation of FUS BBBO, unveiling novel technical challenges and approaches. At present, 3 types of therapeutic ultrasound devices are available for clinical applications: the implanted transducer (eg, CarThera; SonoCloud), the stereotactic frame-based MRI-guided device (eg, Insightec; ExAblate), and the frameless neuronavigation-guided device (eg, NaviFUS).^6^^,^^7^ Each type of methodology employs distinct, specific techniques and requires well-equipped facilities staffed by personnel capable of implementing standardized and replicable protocols. Regardless of the specific hardware employed, significant efforts to refine FUS techniques are crucial for ensuring optimal efficacy, minimizing times, streamlining workflow, and enhancing patient tolerability, participation, and results comparability.^1^

Insights gained from 2 clinical trials, namely BT008 (NCT03551249)^8^ and BT009 (NCT04417088),^9^ wherein MRgFUS (ExAblate Model 4000 Type 2) have informed our approach. In these trials, MRgFUS (ExAblate Model 4000 Type 2) was employed to open the BBB in glioblastoma patients. Building on these insights, we have devised solutions to enhance efficacy, replicability, and tolerability while minimizing the impact of MRI usage, hospitalization, and patient discomfort. This technical note presents our experience of incorporating technical insights and describing the evolution of the workflow, which reflects an ongoing process of learning and development. The final section revisits relevant aspects of the procedure, offering valuable insights that will inform the design of future trials exploring this promising methodology.

## Methods

A review of the procedures of BBBO, performed in the framework of the BT008 and BT009 trials, was conducted at our Institute. The procedures were subjected to retrospective analysis with the objective of elucidating the challenges and evolution of the workflow, with particular attention paid to the learning curve, modifications introduced, duration, volume of BBB opening, and patient comfort.

### Patients Cohort

We reviewed the Patients who underwent BT008 (NCT03551249)^8^ and BT009 (NCT04417088)^9^ trials at Fondazione IRCCS Istituto Neurologico “Carlo Besta” between October 2021 and July 2022. The cohort includes 4 patients: 2 enrolled in BT008 (BT008E-243001, BT008E-243002) and 2 enrolled in BT009 (BT009E-243002, BT009E-243003). Three patients completed 6 cycles of treatment, while one (BT009E-243003) withdrew from the study after 2 cycles for disease progression. All patients were male, aged between 45 and 63 (mean age 55 years). The histological diagnosis was Glioblastoma (WHO 2021 grade 4), at first diagnosis in the case of BT008, and recurrent in the case of BT009. For BT008, patients were enrolled after gross total resection, following the completion of the concomitant phase of the Stupp protocol. In the case of BT009, patients were enrolled at the time of tumor recurrence, following surgical intervention and the Stupp protocol. Further details are available at ClinicalTrial.gov: (NCT03551249)^8^ and BT009 (NCT04417088).^9^

### Clinical Trials

#### Bt008 (Nct03551249)

This is a prospective, multi-center, single-arm study designed to assess the safety and feasibility of opening the blood–brain barrier around the perimeter of the tumor resection cavity using the ExAblate Neuro Model 4000 Type 2 (220 kHz) system with microbubble resonators. Patients eligible for this study must be scheduled to begin the adjuvant TMZ chemotherapy phase of treatment. Participants undergo up to 6 cycles of Exablate BBBO procedures alongside their adjuvant TMZ therapy (150-200 mg/m^2^).^8^

#### Bt009 (Nct04417088)

Phase 1/2, open-label, prospective, multi-center, single-arm study designed to evaluate the safety and feasibility of opening the blood–brain barrier combined with intravenous carboplatin in treating recurrent glioblastoma using the Exablate Neuro Model 4000 Type 2 system with microbubble resonators. Adult subjects with glioblastoma who are scheduled for carboplatin chemotherapy are eligible for inclusion in the study (the planned Carboplatin dose was calculated using the Calvert formula: Total dose (mg) = (target AUC) × (GFR + 25)). Participants undergo up to 6 cycles of Exablate BBBO procedures alongside their carboplatin chemotherapy regimen.^9^

### FUS Equipment

Exablate BBBO procedures are performed with the Exablate Neuro 4000-Type 2 (220KHz) system by Insightec, integrated with a 1.5T MR GE Optima 450W scanner by GE Healthcare. Pre-procedural imaging is acquired on a 3T MR imaging session (Achieva, Philips Healthcare BV, Best, NL, 32-channel Head coil).

### Ethical Aspects

The methodology was approved by the appropriate institutional review board and complied with the ethical guidelines set forth by the Italian Medicines Agency (Agenzia Italiana del Farmaco, AIFA) and national regulations. Informed consent was obtained from all participants in accordance with ethical standards.

## Results

Throughout the 20 procedures performed, an accelerated learning curve was observed. These were the inaugural sessions ever completed in Europe, thus necessitating the navigation of relatively novel challenges. Significant improvements in treatment efficiency were achieved through the implementation of system updates and workflow enhancements, which were designed to address specific challenges. These developments enabled the team to treat larger volumes in less time, ultimately compressing the substantial table time of 3 h to perform both the FUS treatment and the requisite safety and efficacy imaging. To this end, the imaging protocol has been revised by the introduction of in-transducer MRI acquisition within the same Exablate session, thus obviating the necessity for the patient to be relocated for immediate follow-up assessments and providing more expeditious access to subsequent chemotherapy. The following paragraphs present a detailed analysis of the learning curve, outlining the significant challenges encountered and the solutions implemented.

### Patient Comfort

#### Preliminary Phases

**Figure 1.**
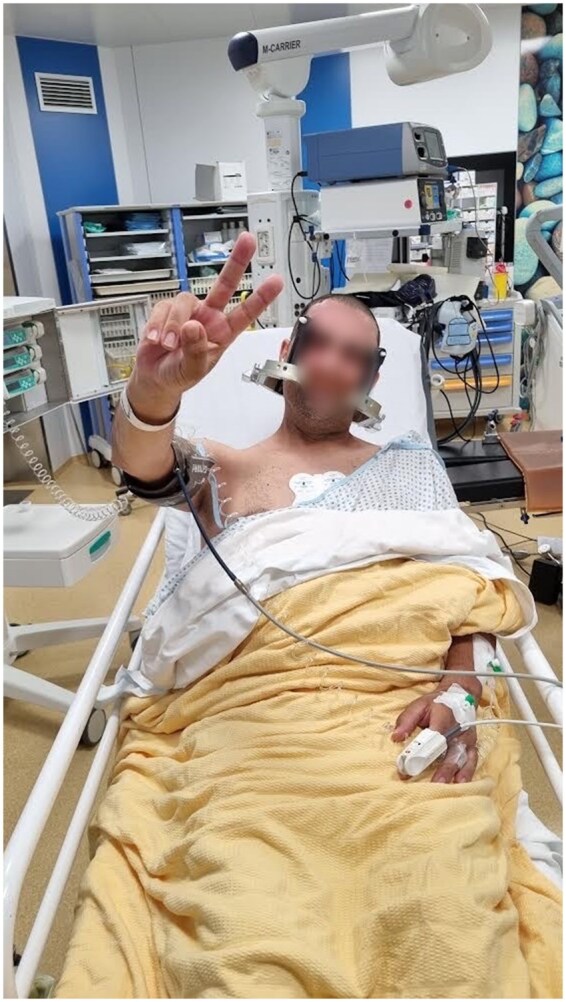
Patient comfort. Great care is dedicated to patient comfort. Pillows and blankets are used to prevent pressure and chilling. Vaseline is used to adhere the membrane to the scalp, preventing water leakage and thus obviating the need for shaving. Compression stockings are applied to prevent deep venous thrombosis in the lower limbs, noise-cancelling earbuds are placed. Local anesthesia is administered at the sites of the stereotactic frame pins to reduce pain. The sedation protocol with dexmedetomidine is implemented, which allows for perfect cooperation and reduces negative perceptions of the procedure, increasing patient tolerance.

As is the case with the BT008 and BT009 trials, FUS procedures entail comprehensive screening evaluations, comprising an assessment of the medical history, physical and neurological examinations, specific laboratory tests, electrocardiography, chest x-ray, and baseline molecular marker analysis. Imaging for planning comprises a high-resolution non-contrast CT (HRCT) scan and MRI to characterize the tumor burden. To minimize stress for patients, the screening procedure has been condensed into a single day of hospitalization, scheduled before the treatment. It has been established that hair shaving represents a significant source of stress. To circumvent this issue, we have implemented a shaveless treatment protocol to preserve hair up to a few centimeters in length which included the application of Vaseline to facilitate the adhesion of the membrane rim to the scalp, thereby preventing water leakage even in the presence of long hair. In the operating room, compression stockings are applied to prevent deep venous thrombosis in the lower limbs, noise-cancelling earbuds are placed to reduce noise perception, and an intravenous (IV) line is established for the administration of fluids, medications, and microbubble resonators during the procedure (Figure 1). It is imperative that patients abstain from food and drink before the procedure, as this reduces the risk of sedation-related complications. Given that the MRI suite is an uncomfortable environment for patients, it is essential to ensure maximum relaxation. This can be achieved by applying pillows or mattress pads to prevent discomfort in the support points and using blankets to maintain body temperature and prevent shivering (Figure 1).

#### Anesthesiology and Sedation Protocol for MRI-Guided Focused Ultrasound

Local anesthetic is administered via subcutaneous injection before the placement of the stereotactic frame at the designated points. The objective of sedation during the FUS procedure is to minimize discomfort while ensuring optimal cooperation from the patient. The patient must remain spontaneously breathing, conscious, and capable of promptly following simple instructions. The patient must remain calm throughout the procedure, tolerating the frame and the prolonged immobilization in the MRI environment. The objective of sedation is to maintain a Richmond Agitation Sedation Scale (RASS) score of 0 or −1 for the entirety of the procedure (Figure 1). To achieve this objective, dexmedetomidine is administered continuously via intravenous infusion by the following protocol:

Monitoring tools utilized in this procedure include the Philips Expression IP5. Three-lead electrocardiographic monitoring, non-invasive arterial pressure, peripheral oximetry for SaO_2_ measurement, and a nasal cannula for etCO_2_ and respiratory rate detection are employed. The intravenous administration of dexmedetomidine is initiated 30 min before the positioning of the frame, through a continuous infusion with an MRI-compatible infusion pump system (Braun) at a dosage of 0.5-1 mcg/kg/h, to achieve a loading dose of 0.25-0.5 mcg/kg over approximately 30 min. The initial infusion rate and loading dose are determined based on the patient’s level of anxiety, with lower dosages initially administered to avoid excessive sedation (Figure 1). The long half-life of dexmedetomidine (approximately 2 h) necessitates a 1-h correction period in the event of an overdose. After the loading dose, the maintenance dose is reduced to 0.2-0.3 mcg/kg/h and modulated on the RASS.^10^ After frame positioning, the patient is then transferred to MRI. If necessary (eg, patient agitated RASS +1), boluses of 10 mcg can be administered intravenously, repeatable after 5 min, while the maintenance infusion continues.

### FUS Procedure

#### Pre-Treatment MRI Protocol

A 3T MR imaging session (Achieva, Philips Healthcare BV, Best, NL, 32-channel Head coil) is conducted 3 days before each Exablate BBBO procedure to exclude the presence of hemorrhages, infarctions, or tumor progression. The imaging protocol (Table 1) includes pre-contrast volumetric T1-weighted fast field echo (T1w-FFE), axial T1w images, volumetric T2w fluid-attenuated inversion recovery (T2w-FLAIR), susceptibility-weighted imaging (SWI), diffusion-weighted imaging, and axial T2-weighted images. Post-contrast (pc) sequences (Gadobutrol 0.1 mL/kg) include volumetric T2w-FLAIR, SWI, and both volumetric and axial T1w-FFE sequences. T1w sequences are acquired at least 15-20 min post-Gadolinium administration.

**Table 1.** Outline of the imaging protocol.

Pretreatment 3T MRI	Pre-sonication in-transducer MRI^b^	Post-sonication in-transducer MRI^b^ ^,^ ^c^
• FFE volumetric T1 w FFE Axial 2 mm T1w • Volumetric T2w-FLAIR SWI • DWI-ADC • FSE Axial 2 mm T2w • Volumetric T2w-FLAIR SWI + *gadolinium* • Axial and Volumetric T1 W + *gadolinium*^a^	• Axial T2-FLAIR SWI^ • Sagittal FSE 1.5 mm T2w • Axial 2 mm SWAN • FSPGR Axial 2 mm T1	• Axial T2w-FLAIR SWI • Axial 2 mm SWAN • FSPGR Axial 2 mm T1 • FSE Axial 2 mm T2w • DWI • Axial 2 mm SWAN + *gadolinium* • Axial 2 mm T2w-FLAIR + *gadolinium* • FSPGR Axial 2 mm T1+ *gadolinium*^a^

a At least 15 min post-gadolinium.

b Constant inclination for axial images.

c Imaging with helmet empty from water.

#### Preplanning and Intra-Operative Pre-Sonication Imaging

**Figure 2.**
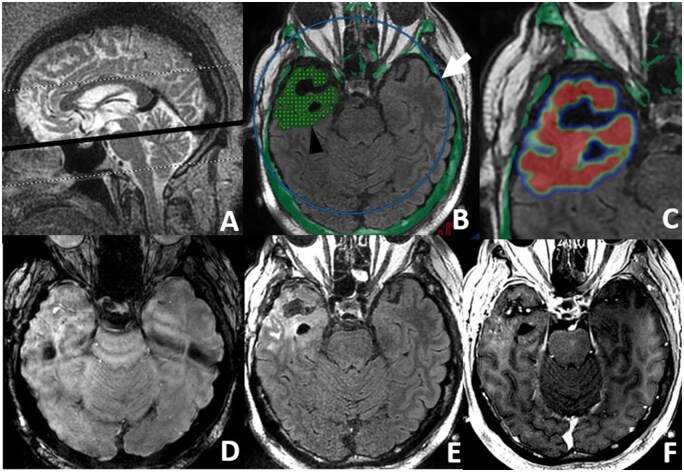
MRgFUS BBBO in a patient during the sixth cycle. “In-trasducer protocol.” (A) Pre-sonication T2w imaging, sagittal plane. The black line represents the reference slice for images B-F showing the predefined inclination maintained during the in-transducer protocol. (B) Targeting on pre-sonication FLAIR images. Two free-shape greeds of maximum 64 subspots covering target area (arrowhead). The treatment envelope, focused on tumoral location is also visible (white arrow). (C) Color-coded cavitation dose map. (D-F) Postprocedural in-trasducer SWAN, pc T2wFLAIR and T1w showing the effects of BBB opening.

Exablate BBBO procedures are performed with the Exablate Neuro 4000-Type 2 (220KHz) system by Insightec, integrated with a 1.5T MR GE Optima 450W scanner by GE Healthcare. For preplanning, on the day of the procedure, 3T FLAIR images are imported onto the Insightec workstation and co-registered with the HRCT acquired in the screening phase. “No-pass” regions are designated over paranasal sinuses to prevent distortions/reflections of the US beams, according to the standard workflow for MRgFUS procedures.^11^ Target areas are determined on 3T T2w-FLAIR by neurosurgeons and neuroradiologists and manually outlined as a reference for intraoperative target placement. Initially, the treatment envelope was not able to cover the entire brain, as a consequence, the stereotactic frame needed to be positioned with the focal point as close as possible to the planned treatment region. However, with the subsequent introduction of the second version of Insightec’s treatment envelope of 150 mm (Figure 2B), enabling near-subtotal coverage of the brain, minimal adjustments are still necessary, only for more peripheral tumors. After securing the silicone membrane/stereotactic frame into the hemispherical dome containing the elements of the Exablate Type 2.0 transducer, the gap between the ultrasound transducer and the scalp is filled with degassed water for acoustic coupling. Pre-sonication MR protocol (Table 1) includes 2D T2w, SWAN (T2*-weighted angiography), FSPRG (fast spoiled gradient echo) T1w, and T2w-FLAIR FS (fat saturated) sequences. Axial scans are conducted with a consistent scan geometry (Figure 2A), thereby ensuring identical inclination and thickness across sequences for geometrically matching planes. Intraprocedural targeting is based on pre-sonication T2w-FLAIR (Figure 2B) for optimal visualization of pathological bright intraparenchymal areas, to maintain pre-selected areas from 3T-preplanning as a reference. Subsequently, the transducer focal point is verified and adjusted post-targeting to concentrate on the desired area. The T2w sequence is employed to identify air pockets within membrane folds, which are then outlined as regions that should be avoided during the subsequent procedure.

#### Sonications

The ultrasound pulse sequences are administered to a maximum of 64 subspots, which are arrayed on a 3-mm-spaced grid. This delineates a free-shaped area in a cyclic sequential pattern, with a repetition time of 1 s. The sequences entail a series of sonications, with an average duration of 108 s each, synchronized with the intravenous infusion of microbubble resonators. Prior to the commencement of the Resonator infusion, a ‘dummy sonication’ is performed to establish a baseline for the acoustic feedback recorded by the Exablate system. Subsequently, the baseline is compared to the feedback from microbubble-enhanced sonications, thereby confirming the activity of the resonator under the influence of focused ultrasound. The burst sonications for each target must commence at least 2 min after the microbubble ultrasound infusion and cease upon the complete infusion administration or after the entire planned volume has been treated. The sonication parameters are modified based on the acoustic feedback. The average power for each treatment sonication was 30 ± 10 Watts, targeting an average volume of 20.5 ± 4.63 cc (Figure 4). SWAN sequences are regularly obtained intraoperatively and immediately post-treatment to identify early BBBO radiological signs,^12^^,^^13^ ruling out hemorrhagic transformation. The sonication phase has an overall average duration of 1 h and 17 min. Moreover, these workflow enhancements, along with technological advancements, have allowed us to reduce the procedure’s average duration from 5 to 3 h of table time, including post-procedural imaging and enhancing the sonicated volume.

#### Post-Operative MRI

Postoperatively, imaging is promptly performed to confirm BBBO and evaluate any potential collateral effects. In the early procedures, we acquired post-procedural imaging using a standard MRI coil for brain imaging. In practice, this required taking the patient out of the MRI, removing the stereotactic frame, and then bringing the patient back into the MRI. This was because the 2-channel coil did not allow for satisfactory image acquisition. To simplify this step, a novel post-sonication MRI protocol (Table 1, Supplementary Materials) has been developed for time efficiency and effective qualitative assessment. Sequences are acquired using the Insightec 2-Ch coil, integrated with the MRgFUS system, immediately after the procedure, retaining the stereotactic frame and without changing to a multi-channel coil or repositioning the patient, a method we termed the “in-transducer protocol.” The water is drained prior to imaging to enhance signal-to-noise ratio (SNR), maintaining the scan geometry consistent with pre-sonication acquisitions.

To confirm MRgFUS-BBBO effects, the protocol includes pre and post-contrast T1w and SWAN sequences (Figure 2D and F), aimed at detecting typical imaging signs of BBB opening, marked by slight, dotted contrast enhancements on T1 and hypointense spots on SWAN sequences within the sonicated regions.^12^^,^^14^ Additionally, pre–post-contrast T2w-FLAIR (Figure 2E) is obtained to assess changes in CSF and perivascular spaces associated with BBBO. The post-sonication imaging duration is approximately 35 min. Overall, the average duration of the patient’s stay in the transducer is 3 h.

#### Drug Administration

After completing the study procedures, the patient is transferred to the ward for chemotherapy. TMZ is administered within 3 h post-procedure (150-200mg/m^2^), and Carboplatin infusion begins within 1 h after BBBO.

#### Early Follow-up

A follow-up MRI at 3T is conducted 1-day post-treatment to exclude side effects such as edema or hemorrhagic transformation and to confirm BBB closure by the disappearance of T1 enhancement phenomena in the treated tissue volume. Furthermore, SWI sequences enabled to confirm the stability or even the reduction of the black spots identified in the post-sonication acquisitions. The scanning protocol replicates that of the screening imaging.

## Discussion

**Figure 3.**
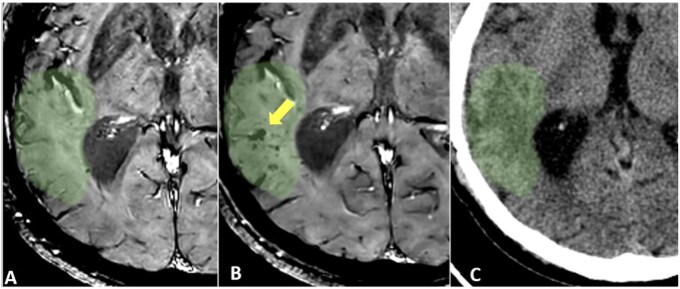
Pre and postsonication imaging in a patient during the first cycle. A-B SWI sequences at pre-treatment MRI (A) and 1-day FU (B) reveal the postoperative appearance of black foci in the sonicated cerebral tissue. (C) 1-day postoperative CT shows the absence of detectable hemorrhagic areas. The green area identify the treated volume, and the red arrow indicates the black spot.

**Figure 4.**
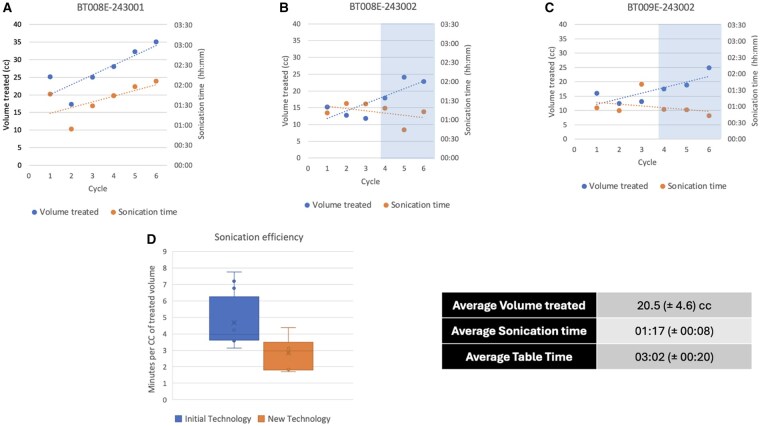
Trend analysis of treated volumes and sonication efficiency for patients who completed the study protocol (6 cycles). The graph of the first patient who completed the protocol (A) demonstrates the steep learning curve and patient management protocol enhancements, allowing to target larger volumes with the patient able to tolerate longer sonication times. (B) and (C) refer to patients who began the cycle later in the learning path. Implementing the improved technology and imaging, combined with all mentioned above, not only allowed a constant increase of treated volume but also a marked tendency of optimizing sonication efficiency, covering larger volumes in shorter times (the blue box in the graph indicated the introduction of the new treatment technology and imaging workflow) (D). No procedure with the all-around optimized workflow exceeded the 3 h mark in patient table time.

**Figure 5.**
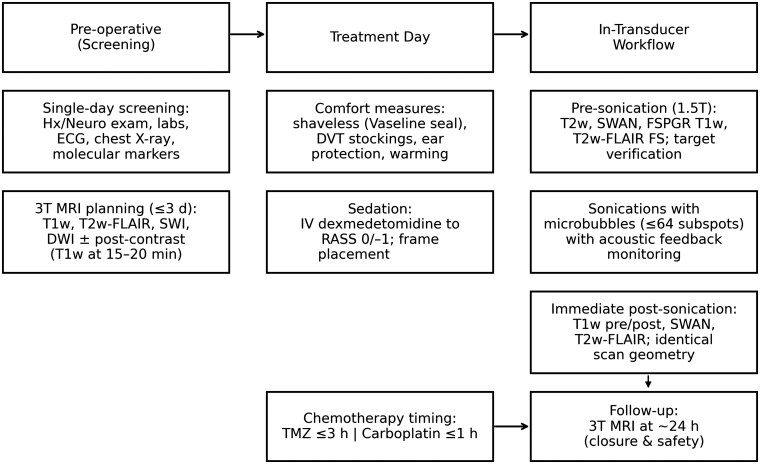
Workflow timeline for MRgFUS-BBBO. The schematic summarizes the streamlined pathway from single-day screening to treatment-day preparation, in-transducer imaging/sonications, and early 24-h MRI follow-up.

This manuscript presents a single-center experience utilizing MRgFUS for BBBO clinical trials in patients with GBM, incorporating technical insights and describing the evolution of a workflow reflective of an ongoing learning curve (Figure 5). Indeed, BBBO represents an innovative and promising methodology that is still in its infancy. This is reflected in the ongoing discovery of potential and practical aspects that lead to the introduction of solutions and refinements. As a result of our experience, we have significantly modified the workflow, reaching the current model presented in this manuscript. A number of pivotal advantages have been identified. Firstly, the interval between the initial sonication and chemotherapy infusion is significantly reduced by shortening both the sonication phase and the immediate post-procedural MR imaging period. Secondly, the quality of the MR images is enhanced due to the absence of motion artefacts, which can occur when the stereotactic frame is removed, and by maintaining the same scan geometry in pre- and post-procedural acquisitions, thus ensuring perfect comparability of images. The protocol developed by our anesthesiologists for the administration of sedation has been demonstrated to be safe, reproducible, and instrumental in facilitating cooperation and optimizing the treatment experience. The efficiency of time in the pre- and procedural phases is primarily achieved through the experience gained by the team and the utilization of the newly introduced technical tools from Exablate. It is noteworthy that this substantial treatment envelope permitted the comprehensive coverage of the cerebral volume, thus obviating the necessity for bespoke helmet fixation or alterations to the transducer focal point. In contrast, with the previous hardware, the smaller treatment envelopes would require precise preplanning for the positioning of the stereotactic frame to ensure proximity of the tumor to the focal point, consequently lengthening the procedure. In addition, the recent availability of a 64-spot grid, compared to the previously used 9- or 32-subspot grids,^15^ enables the treatment of larger target areas, significantly reducing the number of sonications required for the same volume treated and thus decreasing the procedural time from the 2-4 h described in the literature to just 1 h and 17 min^16–18^ (Figure 4). The overall duration of the patient’s stay in the transducer (including the post-procedure imaging) averaged 3 h, which is consistent with durations described in other protocols, without post-procedural imaging.^11^ As illustrated by the graphical representation in Figure 4, the enhancement in the workflow, as previously described, has notably contributed to the expansion of treated volumes. This is due to a combination of factors, including the improvement in the workflow and the patient’s increasing tolerance for longer sonication times. Immediate post-procedural MR controls are crucial to assess BBBO and to detect potential complications.^19–21^ Two-channel (2Ch) system-integrated coils are seldom employed for comprehensive post-procedural imaging due to their restricted SNR.^22^ In contrast, imaging with multi-channel coils on the same or a different scanner provides high-quality images but necessitates patient relocation, removal of the stereotactic frame, and repositioning with the coil replaced. The aforementioned procedure is time-consuming, with a potential duration exceeding 1 h, which consequently delays the patient’s return to the ward for chemotherapy administration. The proposed imaging strategy addresses this issue by optimizing the acquisition of 2-channel images, thereby yielding images of reasonable diagnostic quality. Specifically, bespoke sequence parameters were developed at the local level, motion artefacts were minimized due to the patient’s immobility, and the SNR was enhanced by draining water from the system. Moreover, maintaining the default scan geometry facilitated imaging assessment by achieving perfectly matching slices for pre- and post-contrast acquisitions and among different sequences. Notably, also the interpretation of the effects of BBBO through post-sonication MRI is evolving with experience. A growing body of evidence is supporting the typical findings such as dotted T1 enhancements and black foci in T2* GRE/SWAN sequences in the sonicated areas and their evolution overtime. T1 enhancements usually resolve at the 1-day follow-up, while T2* black foci show a slower tendency to decrease over subsequent MRI follow-ups. While T1 intraparenchymal enhancement is widely recognized as the main MRI finding to confirm BBBO,^1^^,^^5^^,^^23^^,^^24^ the significance of T2* black foci is more debated.^15^ Specifically, although hypointensities on T2*w/GRE-SWAN images usually indicate microhemorrhages or calcifications, many previous studies^15^^,^^23^ confirm, as we observed, their transitory nature with a tendency to reduce at middle and late postoperative follow-ups. For this reason, some authors attribute their nature more to microleakage or venule dilation than microhemorrhages.^15^^,^^23^ Supporting this, the absence of bleeding phenomena in the sonicated tissues showing black GRE T2*/SWAN spots after treatment was previously demonstrated by histological analysis following the surgical excision of treated gliomas.^16^ In our experience, no clinical changes were observed following MRgFUS treatment. Additionally, CT scans, which were occasionally performed during the hospitalization period following treatment, did not detect any evidence of hemorrhagic areas (Figure 3). Although this interpretation may be open to question due to the small size of the black spots, which, even if they were microhemorrhages, could be not detected, in a standard CT scan (Figure 3), the progressive disappearance of these black spots over time and the absence of clinical post-treatment modifications supports the hypothesis that the observed changes are benign and reversible, rather than pathological.

Given the scope of this technical note, we focused on process and feasibility metrics; consequently, the study is limited by the small sample size, the absence of a control/comparator with comprehensive quantitative outcomes (eg, objective BBB-opening metrics or drug concentrations), and its single-center, short follow-up design.

In conclusion, the current stage of development of BBBO and, generally, FUS procedures still require ongoing research and efforts. In this context, our experience could serve as a useful starting point for different settings of FUS treatment to accelerate the learning curve, optimize the procedure, shortening the duration, maximizing the results, and improving the patient’s experience. In the future, studies encompassing larger cohorts of patients, and different methodologies (eg, SonoCloud, NaviFus, Exablate) would yield more robust data to enhance the reliability of the conclusions and the generalizability of the operative protocol.

## Supplementary Material

vdag020_Supplementary_Data

## Data Availability

This study does not involve any sensitive data relevant to the article. All imaging data referenced in the study are stored within the internal Picture Archiving and Communication System of our institution and are not publicly available.
