# Supplementary material for: Focused ultrasound for blood–brain barrier opening in brain tumor patients: Technical nuances
Source: Neurooncol Adv. 2026 Jan 31;8(1):vdag020. doi: 10.1093/noajnl/vdag020 (PMC12978305; doi:10.1093/noajnl/vdag020)
Supplement: vdag020_Supplementary_Data [file vdag020_supplementary_data.zip › Supplementary Materials .docx]

| **Workflow element** | **Earlier/typical approach** | **Our optimized approach** | **Rationale / Impact** |
| --- | --- | --- | --- |
| **Patient preparation** | Hair shaving routinely performed; comfort measures variably applied | **Shaveless** setup using Vaseline seal; pillows/blankets, ear protection, DVT stockings | Minimizes stress; improves tolerance of long MRI sessions. |
| **Sedation & monitoring** | Mixed sedatives; variable targets | **Dexmedetomidine** infusion titrated to **RASS 0/–1** with standard monitoring; small boluses if needed | Preserves cooperation and spontaneous breathing; reproducible, MRI-compatible pathway. |
| **Treatment envelope & targeting** | Smaller envelope; meticulous frame placement to reach periphery | **150-mm envelope** enabling near-total brain coverage; only minor adjustments; targets drawn on 3 T FLAIR | Faster setup; fewer repositioning constraints; simplified pre-planning. |
| **Sonication grid** | 9–32 subspots | **Up to 64 subspots** on 3-mm grid with acoustic feedback | Treats larger free-shape areas with fewer runs; **~1 h 17 min** average sonication time. |
| **Intra/post-procedure MRI** | Patient removed from transducer; frame/coil changes; new positioning | **In-transducer** post-sonication MRI (T1 pre/post, SWAN, T2w-FLAIR), **geometry preserved**, water drained to improve SNR | Cuts motion/repositioning artefacts; immediate confirmation of BBBO; saves time. |
| **Total table time** | ~5 h including imaging | **~3 h** including immediate post-procedure imaging | More efficient session; higher patient comfort. |

**Legends:**

Protocol innovations vs. earlier MRgFUS-BBBO workflows
